# A Multi-Modal Toolkit for Studying Neutrophils in Cancer and Beyond

**DOI:** 10.3390/cancers13215331

**Published:** 2021-10-23

**Authors:** Diana Changirwa, Jared Schlechte, Braedon McDonald

**Affiliations:** 1 Immunology Research Group, University of Calgary, Calgary, AB T2N 4N1, Canada; diana.changirwa@ucalgary.ca (D.C.); jared.schlechte@ucalgary.ca (J.S.); 2 Snyder Institute for Chronic Diseases, Cumming School of Medicine, University of Calgary, Calgary, AB T2N 4A1, Canada; 3 Department of Critical Care Medicine, Cumming School of Medicine, University of Calgary, Calgary, AB T2N 1N4, Canada

**Keywords:** neutrophils, intravital microscopy, organ-on-a-chip, single cell transcriptomics, proteomics, mass cytometry

## Abstract

**Simple Summary:**

Neutrophils are critical immune cells in host defense and maintenance of tissue homeostasis. Studying the complex and diverse functions of these innate immune cells requires a comprehensive toolkit of experimental techniques to elucidate the function and regulation of neutrophils in health and disease. In this review, we discuss key methodologies and their applications in neutrophil research, including in vivo imaging, ex vivo functional assays, and high dimensional single-cell technologies, and how they can be integrated into a multi-modal approach to study neutrophil function in cancer and other diseases.

**Abstract:**

As key effector cells of the innate immune response, neutrophils are rapidly deployed to sites of inflammation where they deliver a payload of potent effector mechanisms that are essential for host defense against pathogens as well as tissue homeostasis. In addition, neutrophils are central contributors to the pathogenesis of a vast spectrum of inflammatory, degenerative, and neoplastic diseases. As our understanding of neutrophils in health and disease continually expands, so too does our appreciation of their complex and dynamic nature in vivo; from development, maturation, and trafficking to cellular heterogeneity and functional plasticity. Therefore, contemporary neutrophil research relies on multiple complementary methodologies to perform integrated analysis of neutrophil phenotypic heterogeneity, organ- and stimulus-specific trafficking mechanisms, as well as tailored effector functions in vivo. This review discusses established and emerging technologies used to study neutrophils, with a focus on in vivo imaging in animal models, as well as next-generation ex vivo model systems to study mechanisms of neutrophil function. Furthermore, we discuss how high-dimensional single-cell analysis technologies are driving a renaissance in neutrophil biology by redefining our understanding of neutrophil development, heterogeneity, and functional plasticity. Finally, we discuss innovative applications and emerging opportunities to integrate these high-dimensional, multi-modal techniques to deepen our understanding of neutrophils in cancer research and beyond.

## 1. Introduction

Neutrophils are powerful immune effector cells that maintain homeostasis in health, fight infections, and participate (for better or worse) across a spectrum of diseases including an expanding role in immuno-oncology. Our understanding of neutrophil biology in health and disease has grown exponentially in recent years, as has our appreciation of the complex bi-directional interplay between neutrophils and other innate and adaptive immune cells, stromal cells, and tissue niches [1,2]. As such, contemporary neutrophil biology is moving towards a systems biology approach to understanding the cellular functions and biological contributions of these critical immune cells in vivo [3]. In addition, research involving neutrophils presents unique challenges compared to other immune cells. Their short life span, inability to maintain in culture or genetically manipulate ex vivo, characteristic motility, and rapidly deployed effector functions requires that neutrophil researchers possess a diverse and versatile toolkit of experimental methodologies to interrogate the tissue-specific, spatial, temporal, cellular, and molecular dimensions of neutrophil functions in vivo.

**Figure 1:**
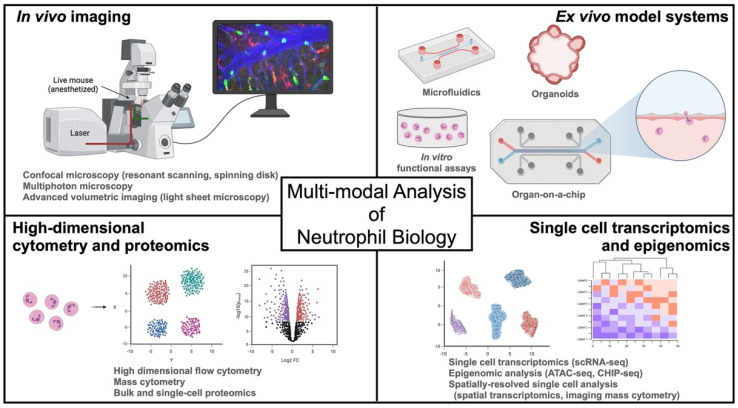
Overview of a multi-modal approach to the study of neutrophils in cancer and beyond. Intravital microscopy is a powerful tool to characterize in vivo cell biology of neutrophil trafficking and function in tissues. Ex vivo assays like laminar flow chambers, microfluidics, and in vitro functional assays, together with emerging technologies of organoids and organs-on-a-chip, are used to understand the dynamic functions of human neutrophils. Deep characterization of neutrophil heterogeneity, plasticity, and functional regulation are facilitated by high-dimensional transcriptomic, epigenomic, and proteomic methodologies, which can also enable a systems biology approach to the study of neutrophils in vivo.

Herein, we review commonly used and emerging technologies in neutrophil research, including in vivo (intravital) imaging, innovative ex vivo systems to study neutrophil function, and powerful single-cell multi-omics applications (Figure 1). We highlight key discoveries that have been made using a multi-modal approach and propose a framework for the investigation of neutrophil functions within a systems biology context that can be employed to study neutrophils in cancer and beyond.

## 2. Seeing Is Believing—In Vivo Imaging of Neutrophil Trafficking and Function

The function of neutrophils is directly linked to their ability to traffic from the blood into tissues and migrate to sites of infection and inflammation [4]. Therefore, studying the locomotion of neutrophils is critical to understanding their function in vivo. The most powerful research tool available to characterize neutrophil trafficking in vivo is intravital microscopy (IVM), which involves surgical exposure of an organ of interest (in a live anesthetized animal) and direct visualization with a microscope to observe neutrophil behavior in blood vessels and tissues [5]. Within the field of immuno-oncology, IVM has proven particularly powerful to study the trafficking and function of neutrophils within tumors as well as their contribution to cancer metastasis [6,7,8,9]. In this section, we provide a primer overview of the most common contemporary IVM modalities (confocal and two-photon imaging) and explore emerging IVM technologies that will expand our ability to discover new aspects of neutrophil biology in vivo.

Imaging neutrophils, their trafficking, and effector mechanisms within diverse tissue microenvironments in vivo requires highly versatile microscopy platforms. Key characteristics that are needed for advanced IVM include fast image acquisition (to capture the rapid activities of neutrophils in real-time), high resolution (to observe cellular and subcellular details), volumetric coverage (to study the 3D architecture of vessels and tissues), and multi-color capabilities (to simultaneously observe multiple fluorescent markers on neutrophils and other cells). There is an inherent trade-off between these important parameters and the unwanted side effect of phototoxicity, which includes photobleaching of fluorophores as well as biological toxicity caused by excessive photon energy on cells and tissues [10]. Furthermore, commonly used IVM platforms each have unique advantages and disadvantages with respect to speed, resolution, signal-to-noise, volumetric coverage, fluorescence spectrum, and phototoxicity that must be considered when choosing the most appropriate IVM platform for one’s experiments (Table 1). Lastly, researchers must also consider the substantial differences in cost and expertise required to operate different IVM platforms.

### 2.1. Confocal Microscopy

Confocal microscopy uses laser light together with pinholes to perform high-resolution imaging of fluorophores within tissues by minimizing out-of-focus (and out-of-spectrum) fluorescent signals. The ability to simultaneously or sequentially excite tissues with a range of laser light wavelengths, together with optical and digital methods to separate emitted light spectra, has allowed researchers to achieve high-resolution multicolor imaging of cells and tissues in vivo. An important limitation of standard point-scanning confocal is the slow acquisition speed (commonly 1–5 frames per second at 512 × 512 pixels), which is typically too slow to visualize real-time activities of cells in the bloodstream, or rapid events that unfold in live tissues [11] (Table 1). However, advances in scanning systems, notably the application of resonant-scanning and spinning-disc confocal technology, have dramatically increased the speed of acquisition. For example, resonant scanning systems that operate at 8000 Hz, as well as spinning disk systems enable imaging of in vivo cell dynamics at 30 frames per second or higher. The increased speed of image acquisition achieved by these confocal systems allows researchers to maintain excellent resolution and low signal-to-noise ratio, while being able to visualize very rapid events in neutrophil function (such as the initial tethering and rolling steps of neutrophil recruitment, or rapid cell–cell interactions such as those between neutrophils and platelets) [12,13,14,15,16,17]. Lastly, an important additional benefit of confocal IVM is the (relatively) lower cost and ease of use compared to multiphoton and other advanced microscopy platforms.

Key limitations of confocal imaging may include a requirement for higher doses of laser light to excite fluorophores and the resulting photobleaching and phototoxicity, challenges separating fluorophore signals from tissue autofluorescence, and perhaps most importantly, limited volumetric coverage (primarily due to limited ability to image deep into tissues) [11,18]. Studying neutrophil dynamics within tissues in vivo requires analysis of their locomotion in four dimensions (x, y, z, and time). To achieve the depth of imaging in the z-dimension, confocal microscopes require axial scanning of multiple focal planes (z-stacks) with subsequent digital reconstruction of 3D images. However, light scattering and out-of-focus fluorescence emission limit the depths that can be achieved by conventional confocal imaging. Therefore, confocal IVM is best applied in organs that require imaging at depths less than ~50 μm, such as organs where neutrophil recruitment and function can be observed in close proximity to the surface (e.g., skin [19], liver [20], lung [21,22], skeletal muscle [15,23], brain/meningeal vasculature [24], and others).

### 2.2. Multiphoton Microscopy

The development of multiphoton IVM has enabled high resolution in vivo imaging at greater depths and with lower phototoxicity compared to standard confocal imaging [18,25]. Multiphoton microscopy is based on the principle that fluorophores can be excited by the sum of two low-energy (longer wavelength) photons when they simultaneously converge upon the fluorophore [25]. This ability to utilize longer wavelength light allows for deeper penetration of photons into tissues, and the requirement for spatial convergence of two photons at a very precise focal plane yields less out-of-focus fluorescence excitation, photobleaching, and phototoxicity. This technology has allowed researchers to visualize immune cell function within intact organs that were outside the depth of standard confocal, such as the spleen, lymph nodes, brain, tumors, and deep within other solid organs [26,27,28,29]. Furthermore, multiphoton imaging provides added benefits such as label-free second harmonic detection of organized tissue structures, such as collagen architecture [30].

Multiphoton imaging involves more complex optical hardware than standard confocal microscopes, including tunable titanium: sapphire lasers that function in the near-infrared range. The cost and physical space required for these systems can limit their availability, and also limit the number of excitation wavelengths (and thereby fluorophores/colors) that can be imaged simultaneously. However, the superior depth of imaging has made multiphoton IVM an indispensable tool in the armamentarium of neutrophil biologists.

### 2.3. Challenges and Emerging IVM Technologies

As technical advances in IVM have expanded the resolution, depths, and speed at which in vivo fluorescence imaging can be performed, it has allowed researchers to uncover new dimensions of neutrophil function in vivo. This includes advances in microscopy technology as well as mouse models and imaging applications. For example, recent studies combining advanced IVM imaging with transgenic reporter mouse models have led to the discovery and characterization of previously unrecognized trafficking behaviors of neutrophils at the site of inflammation such as reverse transendothelial migration (rTEM, the movement of neutrophils out of inflamed tissues back into the bloodstream) [31,32,33], as well as novel mechanisms that restrain neutrophil infiltration to limit excessive inflammation involving endothelial cell autophagy [23]. In addition, there have been a number of recent advancements in microscopy technology that allow much larger volumes of tissue to be imaged simultaneously, allowing researchers to study organ-level spatial regulation of immune function. Digital image stitching is an established method that constructs large volumetric images from tile scans of multiple smaller fields of view. For example, volumetric analysis of the inflamed liver using tile scanning and digital image stitching was used to uncover important spatial regulation of neutrophil recruitment, endothelial function, and macrophage localization within the liver microcirculation that was not appreciated in previous studies due to the constraints of analyzing individual fields of view [34]. Although powerful, the digital image stitching approach to volumetric analysis is limited by a relatively slow speed of acquisition, because it takes a long duration for the microscope to sequentially acquire multiple fields of view. To overcome this problem, an emerging area of IVM research seeks to apply simultaneous volumetric imaging methodologies such as light-sheet microscopy for real-time in vivo imaging [35]. Most commonly used to image fixed and cleared tissues ex vivo, light-sheet microscopy has seen many recent advances in 3D scanning and optical methods for enhanced penetration depth that now allows for use with intravital imaging in live tissues. For example, Wang et al. recently reported a near-infrared excitation light-sheet microscopy tool that was used for volumetric multi-color imaging of the brain microvasculature in anesthetized mice through an intact scalp and skull to a depth of ~750 μm, as well as imaging of lymphocyte behavior within intact tumors in mice without the need for any surgical manipulation of tissues [36,37].

Researchers now seek to move beyond simply observing cellular behaviors with IVM and strive to visualize subcellular and molecular activity within live cells in vivo. However, imaging at a subcellular resolution over long periods of time in vivo continues to be challenging due to the consequences of phototoxicity and photobleaching. To overcome these limitations, Wu et al. recently reported a ground-breaking approach using a novel technology called DAOSLIMIT (digital adaptive optics scanning light-field mutual iterative tomography) [38]. Compared to either spinning disk or two-photon IVM, DAOSLIMIT enabled long-term (many hours) 3D IVM at ultra-high resolution with less phototoxicity and more stable fluorescence. Using this technology to interrogate neutrophil trafficking in the liver microcirculation of mice, the investigators observed that migrating neutrophils deposited small cell fragments (called “migrasomes”) as they crawled through the liver sinusoids in vivo [38]. While the functional implications of these migrasomes remain to be fully elucidated, studies like this highlight the need for IVM modalities that allow for stable long-term imaging at a subcellular resolution to understand the nuances of neutrophil function in vivo.

Lastly, the study of neutrophils with IVM has benefitted from developments in fluorescent reporters and biosensors that can be used to perform functional neutrophil imaging in vivo. For example, the generation of lineage-specific neutrophil fluorescent reporter mice has improved the specificity of in vivo neutrophil imaging and has been further refined by the development of optogenetic methods such as photoactivatable GFP neutrophils for fate mapping experiments [33,39,40]. In addition, advances in fluorescent biosensors have made it possible for IVM research to move beyond simply visualizing the structure and activity of cells, towards imaging molecular mechanisms of effector functions in vivo such as NETs production [13], protease activity [41], oxidative bursts [42], and phagosome acidification [42], as well as interactions with other biological systems such as the coagulation cascade [14]. While these advances have led to exciting new discoveries of neutrophil and other immune cell functions in vivo, a key limitation of current IVM technology is the relatively limited number of fluorophores/colors that can be imaged simultaneously, thereby restricting the ability to apply a “systems biology” approach using IVM. Therefore, further developments are needed towards multiplexing and label-free imaging to expand the number of markers that can be imaged simultaneously to study the complex interactomes of cellular and molecular events that unfold during immune responses in vivo.

## 3. Understanding Human Neutrophils in Context—Ex Vivo Systems to Study Human Neutrophil Function in Vascular and Tissue Microenvironments

Unlike animal models, intravital imaging in humans is quite limited, and only possible within vasculatures that can be accessed non-invasively such as the sub-lingual or buccal mucosa, retina, or colonic mucosa, or invasively during surgical procedures [43,44]. Therefore, human neutrophil research remains highly dependent on ex vivo model systems to study the cellular and molecular mechanisms of migration and function in the human context. In particular, systems that model the intravascular microenvironment such as laminar flow chambers, often coated with cultured endothelium or immobilized proteins, have led to landmark discoveries of the mechanisms that control neutrophil tethering, rolling, adhesion, transendothelial migration, and chemotaxis [45,46,47]. However, prototypical ex vivo systems like flow chambers, transwell assays, and in vitro functional assays are unable to replicate the complex and nuanced context of vascular and tissue microenvironments that neutrophils must navigate in vivo [48]. Therefore, contemporary ex vivo assays aim to incorporate previously overlooked biophysical, cellular, and physiological parameters to the study of human neutrophil biology.

### 3.1. Microfluidics

In vivo imaging of mouse organs has revealed that a substantial amount of tissue-level neutrophil migration and function is actually conducted within the microvasculature rather than the extravascular/parenchymal space [49]. This has prompted researchers to develop ex vivo systems to model microvascular architecture and fluid dynamics using microfluidic chambers. The ability to control conduit diameter, circuitry, surface conditions, fluid dynamics, and chemoattractant gradients has enabled researchers to decipher how neutrophils regulate polarization, adhesion microdomains, and to discover new mechanisms in the molecular adhesion cascade that controls neutrophil egress from the blood [50]. For example, a recent study by Wang et al. used bifurcating microfluidic channels to understand how neutrophils make decisions on which path to travel when they encounter vessel branches [51]. Interestingly, the authors found that chemoattractant gradients and hydraulic resistance within individual channels are sensed by trailing neutrophils causing them to take alternate routes, thereby allowing a more efficient “flow of traffic” through the vasculature [51].

### 3.2. Organoids

The development of self-organizing 3D organoids from stem cell precursors has been widely utilized to model human biology in vitro to investigate genetic disorders, degenerative diseases, cancer, infections, and other pathologies [52]. In addition, organoid systems have created a new frontier in precision drug development that holds promise for personalized medicine through the ability to create complex multicellular environments from patient-derived samples. The evolving ability to model complex and dynamic multi-cellular systems in vitro, from host-microbiota interactions to immune–tumor interactions, has proven a powerful tool to study human disease [53,54,55]. Currently, organoid systems remain underutilized in the field of neutrophil biology but hold great potential to understand organ-specific neutrophil responses. For example, Sachs et al. recently developed human airway organoids to model a range of diseases including cystic fibrosis, lung cancer, and pulmonary infections, and used this system to study neutrophil trafficking in model airways using a co-culture approach [56]. Perhaps the greatest barrier to the use of organoid systems to study neutrophil–tissue interactions currently is the lack of vascularization and perfusion, although this is an active area of research with promising developments towards modelling perfused organoids [57,58,59].

### 3.3. Organs-on-a-Chip

An innovative and emerging approach to ex vivo modeling of human neutrophil responses within tissue microenvironments combines microfluidics with 3D multi-cellular culture to yield “organ-on-a-chip” devices that replicate functional organ units [60]. Improving upon traditional flow chamber systems, first-generation organs-on-a-chip model organ-specific microvasculature, in which microfluidic channels are coated with organ-derived endothelial cell monolayers. This methodology has been used to study neutrophil trafficking and host–pathogen interactions in lung microvasculature by Lee et al., who developed a human lung microvascular system using pulmonary endothelial cell-coated microchamber slides to study the interaction between neutrophils and pathogens (*Candida albicans*) under physiologic flow conditions [61]. Next-generation organ-on-a-chip devices aim to recapitulate entire functional organ units. For example, lung-on-a-chip systems have been developed that replicate the blood–air interface using flexible membrane microfluidics devices as a scaffold to culture “breathable” alveoli (i.e., stretchable alveolar epithelial structures that can be ventilated) adjacent to fully endothelialized vascular conduits through which blood/cells can be perfused [62]. Overall, organ-on-a-chip technology represents a promising approach to studying human neutrophil responses within the context of tissue-specific microenvironments in a wide range of oncologic and non-oncologic diseases.

Lastly, an exciting forefront in organ-on-a-chip technology is the ability to utilize bioprinting (3D printing of cells and matrix scaffolds) to accelerate the speed and scalability of these powerful in vitro model systems. As recently reviewed by Yu and Choudhury, the construction of organ-on-a-chip systems using automated bioprinting methods enables highly reproducible and efficient high-throughput testing using these powerful in vitro organ systems [63]. Such technology may help to make these methods more widely available and standardized for use in future neutrophil research.

## 4. A Renaissance in Neutrophil Biology—High-Dimensional Multi-Omics Analysis of Neutrophils

Single-cell approaches to transcriptomic, epigenomic, and proteomic analysis have re-defined many fundamental concepts of neutrophil development, plasticity, and functional heterogeneity [64,65,66,67]. As these methodologies continue to become more accessible and affordable, their application in neutrophil biology has expanded. However, due to a number of unique challenges posed by neutrophils, the use of high-dimensional single-cell techniques in neutrophil research remains comparatively underrepresented in the literature. Therefore, much remains to be discovered about the functional landscape of neutrophils using these approaches.

### 4.1. Transcriptomics

Single-cell RNA sequencing (scRNA-seq) has revolutionized our understanding of immune cell development, maturation, and functional plasticity [68]. However, neutrophil biologists have historically placed little emphasis on transcriptional regulation of neutrophil functions, since mature circulating neutrophils display relatively low transcriptional activity, and their cardinal effector functions (e.g., phagocytosis, oxidative bursts, NETs, proteolysis) are largely regulated at post-transcriptional and post-translational levels [1,2]. In addition, neutrophils are technically challenging to study in vast scRNA-seq datasets (especially those derived from in vivo tissues) due to their relatively low transcript counts in sequencing datasets that render these cells susceptible to filtering algorithms based on read count thresholds. Furthermore, prototypical markers used to identify neutrophils (such as Ly6G in mice and CD66b in humans) are insufficiently expressed at the mRNA level to be used for identification, making the (seemingly simple) task of recognizing neutrophils within complex samples somewhat challenging. Many studies to date have overcome this limitation by pre-sorting or enriching neutrophils from homogenized tissues (using magnetic or florescent cell sorting prior to scRNA-seq), or by methods such as CITE-seq (cellular indexing of transcriptomes and epitopes by sequencing) that incorporates oligomer-tagged antibodies to enable representation of protein markers in the sequencing dataset (e.g., classical cell surface markers), as well as advancements in downstream bioinformatic analyses [66,69]. With these advances, single-cell transcriptomic analysis of neutrophils has become more accessible using conventional high throughput sequencing platforms, and as a result, there has been a marked increase in the application of this methodology in recent years [64,65,66,67,69,70,71].

### 4.2. Epigenomics

The recognition that neutrophils exist in transcriptionally distinct subsets and maturation states has prompted researchers to investigate the role of chromatin accessibility and transcription factor regulation in neutrophil development, plasticity, and function. Epigenomic methodologies, such as the assay for transposase-accessible chromatin with sequencing (ATAC-seq) and chromatin immunoprecipitation and sequencing (CHIP-seq), have illuminated neutrophil-specific factors of gene expression and regulation at the epigenetic level [71,72,73]. Most impressively, these techniques were recently used to challenge the dogmatic view of neutrophils as terminally differentiated effectors by two studies that combined transcriptomics and ATAC-seq to show that neutrophils acquire tissue-specific imprinting upon egress from the bone marrow into blood, and again upon recruitment into tissue microenvironments [66,67]. Driven by chromatin remodeling and promoter access by a stereotyped set of transcription factors, neutrophils were shown to rapidly acquire tissue-specific gene expression programs when they entered different organs throughout the body [66,67]. These discoveries have revolutionized our understanding of neutrophils as cells that display surprising plasticity driven by tissue- and context-specific epigenetic imprinting. However, single-cell approaches to epigenetic analysis still face challenges of scalability and cost [74].

### 4.3. Proteomics

In addition to transcriptomics, our emerging understanding of neutrophil heterogeneity and distinct subsets has been propelled by high-dimensional cytometry and proteomic analyses. Building upon the concept of flow cytometry, time-of-flight mass cytometry (CyTOF) uses heavy metal conjugated antibodies to interrogate surface and intracellular protein expression on single cells [75]. Escaping the constraints of fluorescence detection (limited fluorophores, spectral overlap), the use of mass spectrometry to detect metal-conjugated antibodies vastly increases the number of markers that can be simultaneously analyzed on single cells, while also allowing for barcoding and multiplexing of samples. Furthermore, the advent of reliable methods to cryopreserve cells for CyTOF analysis has proved particularly beneficial for human studies that require longitudinal sample collection followed by batched analysis [76,77,78]. As a result, CyTOF has been used extensively to characterize the landscape of the innate and adaptive immune systems in health and disease. More recently, mass cytometry has become a common tool to study neutrophil phenotypic and functional heterogeneity in development and homeostasis [79], infection [70,80,81], inflammatory diseases [82], as well as cancer [83,84].

In addition to cytometry-based proteomics, bulk cellular proteomic analysis by mass spectrometry has been used to characterize post-translational mechanisms of neutrophil function, and to define the protein composition and dynamics of key effector mechanisms like NETs [85]. Recently, Adrover et al. conducted an elegant analysis of diurnal changes to the neutrophil proteome and uncovered circadian regulation of neutrophil granules, their protein composition, and the ability to produce NETs [86]. Characterization of the proteome of neutrophils isolated at different times of day revealed stereotyped diurnal expression of neutrophil granule proteins, including prominent changes in NETs-associated proteome, and progressive loss of granule proteins involved in inflammation and NETs [86]. This disarming of effector functions was intrinsically regulated within the neutrophil pool by the circadian rhythm-associated protein Bmal1 and the chemokine receptor CXCR2, and protected against excessive inflammation in a murine model of acute lung injury [86]. Interestingly, the diurnal oscillations of the neutrophil proteome showed poor correlation with the transcriptome, again highlighting the importance of a multi-modal approach to discover new aspects of neutrophil function and regulation in vivo.

### 4.4. Spatially-Resolved Single Cell Analysis

As noted above, the function of neutrophils in vivo is inherently linked to their ability to traffic from the blood into tissues and migrate within tissues to localize their assault at the appropriate site. Not only is there critical spatial regulation at the initiation of neutrophil responses, but recent evidence has even demonstrated that ongoing neutrophil accumulation at sites of inflammation is a self-regulating process based on the spatiotemporal organization of cell swarming in tissues [87]. Therefore, studying neutrophil responses in tissues using conventional single-cell techniques that ignore spatial architecture (due to the requirement to dissociate tissues into single-cell suspensions) may lead researchers to miss important information about how neutrophils function in vivo. Fortunately, methods have been developed to perform high-dimensional single-cell characterization of cells within intact tissues that incorporate spatial orientation and tissue architecture into the analysis. Named by *Nature Methods* as the “method of the year for 2020”, spatially resolved transcriptomics involves single-cell RNA-seq of tissues with preserved architecture (typically tissue slices), either through microdissection or in situ hybridization approaches [88]. In addition to transcriptional analysis, spatially resolved proteomics has also become possible using mass cytometry imaging modalities such as multiplexed ion beam imaging (MIBI) and imaging mass cytometry (IMC). Very much a field in development, widespread application of these methodologies is currently limited by the cost and requirements for specialized expertise and infrastructure. However, spatially resolved single-cell analysis is proving very powerful to add new dimensions to the systems biology of cancer as well as inflammatory and immune diseases. As an example, these powerful methodologies were recently employed to create the first spatially resolved cellular atlases of the pulmonary immune response to SARS-CoV-2 infection, unveiling critical details of the immune response in COVID-19 [89,90].

Lastly, an exciting frontier for spatially resolved single-cell -omics is the ability to perform transcriptomic analysis in conjunction with live in vivo imaging. Still in its infancy, this approach is particularly appealing for studying neutrophils, as cellular mobility and locomotion are *sine qua non* with their function in tissues. Current approaches include the use of intravital microscopy to visualize cells of interest in vivo, tag them in situ using a photoactivatable-GFP reporter system, followed by FACS sorting of imaged GFP-positive cells for further analysis by scRNA-seq [91]. As an active area of research and development, live in vivo scRNA-seq holds great potential to understand the impact of neutrophil heterogeneity (subset, polarization states) on cell trafficking and effector responses within tumors and metastatic niches in vivo.

## 5. Conclusions

### A New Way Forward—Integrated, High-Dimensional, Multi-Modal Approaches to Study Neutrophils in Cancer and Beyond

With our evolving appreciation of the heterogeneity, plasticity, and context-dependent functions of neutrophils in vivo, it is evident that the vanguard of neutrophil research requires a multi-modal approach. The contemporary neutrophil biologist needs to incorporate a spectrum of high-dimensional multi-omics technologies coupled with in vivo cell biology to comprehensively understand the functions of neutrophils within tumor microenvironments. Furthermore, the power of these techniques is further amplified by the growing availability of transgenic mouse models for the study of neutrophils in vivo (lineage-specific reporters, inducible depletion, optogenetic manipulation, and others—recently reviewed by Stackowicz and colleagues [92]). Given that neutrophil development, maturation/polarization, and function are regulated at multiple levels (epigenetic, transcriptional, and post-transcriptional), it is increasingly important to integrate a spectrum of genetic, proteomic, and dynamic functional analyses into neutrophil studies. Furthermore, studying neutrophils in isolation yields an incomplete picture of their role in disease, whereas adopting a systems biology approach that incorporates the bi-directional interaction between neutrophils and other immune, stromal, and malignant cells would provide a more comprehensive understanding of their role in cancer pathogenesis. Taken together, this highlights the importance of applying a multi-modal approach to understand the complete landscape of molecular and cellular regulation of neutrophil functions and their roles in cancer and other inflammatory and infectious diseases.

This approach may be particularly well suited to the study of neutrophils within the complex and dynamic microenvironments of tumors in vivo. Building upon our emerging understanding of neutrophil heterogeneity within the tumor niche, further research is required to uncover the mechanisms that regulate neutrophil plasticity and function in response to malignancy in vivo, and the spatio-temporal mechanisms that control their trafficking and locomotion within tumors. Such questions are well suited to the use of single cell multi-omics approaches, particularly those that afford a spatially resolved assessment of neutrophils within the tumor architecture. In combination with high-dimensional IVM in mouse models, these techniques will enable a deep understanding of the protective and pathological functions of neutrophils and their dynamics within tumor niches. In addition, ex vivo tumor organoids or tumor-on-a-chip systems can be employed to translate discoveries to the human context. Most excitingly, these methods could be seamlessly adapted to screen and evaluate novel drug candidates for immunotherapies that target neutrophil–tumor interactions. Together, this multi-modal toolkit will allow scientists to uncover new depths of neutrophil biology to advance translational research in immuno-oncology.

## Figures and Tables

**Table 1:** A comparison of common intravital microscopy platforms.

Imaging Modality	Key Advantages	Key Disadvantages
Epifluorescence	Inexpensive Accessible	Often lower resolution More rapid photobleaching Slower acquisition (lack of simultaneous multichannel imaging)
Point-scanning confocal	Accessible technology in most institutions	Slow acquisition speed Higher phototoxicity to live cells and tissues
Resonant-scanning confocal	High speed of image acquisition	Often requires averaging of multiple laser passes to achieve adequate signal (which reduces speed and increases phototoxicity)
Spinning-disk confocal	High speed of image acquisition Lower phototoxicity/bleaching	Limited depth of tissue penetration (similar to other confocal modalities)
Two-photon	Deeper tissue penetration Less out-of-focus fluorescence signal	Higher cost More technically demanding Often slower acquisition speed
